# Analysis of pretreatment factors associated with stability in early class III treatment

**DOI:** 10.1186/s40510-021-00371-7

**Published:** 2021-07-19

**Authors:** Yasuko Inoue, Toru Deguchi, James K. Hartsfield, Wakako Tome, Noriyuki Kitai

**Affiliations:** 1 Inoue Orthodontic Clinic, Osaka, Japan; 2 Division of Orthodontics, College of Dentistry, Ohio State University, 4088 Postle Hall, 305 W. 12th Ave, Columbus, OH 43210 USA; 3 Division of Orthodontics, Department of Oral Health Science, College of Dentistry, University of Kentucky, Lexington, KY USA; 4 Department of Orthodontics, School of Dentistry, Asahi University, Gifu, Japan

**Keywords:** Early class III, Stability, Mounted cast models, Cephalometric, Pretreatment factors

## Abstract

**Background:**

The purpose of this study was to identify pretreatment factors associated with the stability of early class III treatment, since most orthodontists start the treatment with their uncertain hypotheses and/or predictions. Subjects consisted of 75 patients with a class III skeletal relationship (ANB < 2° and overjet < 0 mm) who had been consecutively treated with rapid maxillary expansion and facemask and followed until their second phase treatment. The patients were divided into two groups according to whether they showed relapse in follow-up. The stable group maintained their positive overjet (*n* = 55), and the unstable group experienced relapse with a zero or negative overjet (*n* = 20). Two general, three dental, and 13 cephalometric pretreatment factors were investigated to determine which factors were associated with stability.

**Results:**

Sex, pretreatment age, and anteroposterior functional shift, which were hypothesized as associated factors, were not related to the stability of early class III treatment. Significant differences were detected between the two groups in the horizontal distance between the maxillary and mandibular molars in centric relation. Cephalometric variables, such as the mandibular length (Ar-Me), Wits appraisal, SN to ramus plane angle (SN-Rm), gonial angle, incisor mandibular plane angle (IMPA), and Frankfort plane to mandibular incisor angle (FMIA) showed significant differences between the groups. The horizontal distance was the most influential factor by logistic regression analysis.

**Conclusions:**

Hypothesis (related to sex, age, functional shift) were rejected. Several cephalometric factors related to the mandible were associated with stability. The horizontal distance between the maxillary and mandibular molars in centric relation was the best predictor of early class III treatment relapse.

## Background

Determining which factors are associated with the stability of early class III treatment remains a major concern for orthodontists. Several studies have been conducted to identify these factors [1–8]. Fudalej et al. [9] concluded in their systematic review that no universal and precise predictor of early class III treatment outcomes has been revealed because of the marked variation in approaches. Recently, it was also reported that no method or factor can predict the long-term success of orthopedic treatment for skeletal class III malocclusion [10]. Thus, it is still essential to identify these factors, especially in the Asian population, which includes a high proportion of class III cases [11].

This study was conducted to verify several hypotheses proposed from the literature [1–16]. The first hypothesis is the outcome for male patients is worse than that for females because of the male’s greater pubertal growth [13]. The second hypothesis is cases started earlier are more stable because maxillary protraction headgear is more effective in younger patients [14, 15]. The third hypothesis is cases with an anteroposterior functional shift are more stable because they are a pseudo-class III and are thought to be easier to treat [16]. In addition, the last hypothesis is that some factors may be related to post-treatment stability.

## Methods

The study protocol was reviewed and approved by the ethics committee for research of the Asahi Dental University of Japan (IRB No. 28001).

Original sample of 96 consecutive patients with a class III skeletal relationship (ANB < 2° and overjet < 0 mm) who had received combined rapid maxillary expansion (Hyrax-type) and facemask therapy and were followed until their second phase treatment in a private practice. Seventy-five patients (38 males: age, mean ± SD, 9.17 ± 0.29 years; and 37 females: age, 8.31 ± 0.24 years) who satisfied the following inclusion criteria were selected for the final analysis: (1) no dental agenesis, (2) no open bite, (3) no lateral crossbite, (4) permanent central incisors present, and (5) no craniofacial anomaly or syndrome. No subjects were excluded on the basis of the stability of early treatment.

According to a previous study [17], the difference between the mean values of the molar relationships between the stable and relapse groups was 4.1 (SD 3.72) to 5.6 (SD 2.84). Assuming a type I error of 5% and a type II error of 20%, the number of subjects required in one group is 4–11 or greater, and by converting the ratio of the numbers in the stable and unstable groups in this study from 3–8.34 to 7–9.46, we obtained a sufficiently reliable sample size. All patients were Japanese, and informed consent was obtained from all participants.

The range of the total expansion was 3–5 mm (2 turns/day). Patients were also given a facemask when the expander was placed. Elastics were attached to the anterior region of the maxilla, in a downward and forward direction (as parallel as possible to the occlusal plane), producing 200 to 250×*g* of force per side. The patients were instructed to wear the facemasks for at least 12 h per day. Patient cooperation was checked with a chart reporting the length of time the facemask was worn daily. The appliances were worn until a positive overjet was achieved. The mean time elapsed before a positive overjet was obtained was 11.16 ± 2.97 months.

After the first phase treatment, wrap-around retainers were provided, and all patients were followed every 4–6 months until their second phase treatment, at least 1 year after the annual peak growth velocity began to decrease (mean age 14.89 ± 0.84 years, males 15.09 ± 0.09 years, females 14.69 ± 0.14 years). The annual peak growth velocity data were obtained using height charts with measurements performed at the patients’ schools. Additionally, the cervical vertebral maturation (CVM) method was used; all patients at this timepoint were either CVMS IV or V [18].

At this time point, the patients were classified into two groups according to whether or not they had relapsed. Patients who maintained a positive incisor overjet before their second phase treatment were classified into the stable group (*n* = 55), and patients who had relapsed to edge-to-edge or a negative incisor overjet were classified into the unstable group (*n* = 20). Pretreatment data of these patients were evaluated to find the factors associated with stability as follows: (1) sex, (2) pretreatment age, (3) presence or absence of anteroposterior functional shift, (4) horizontal and vertical distances between molars on mounted models, and (5) 13 cephalometric data measurements.

**Fig. 1:**
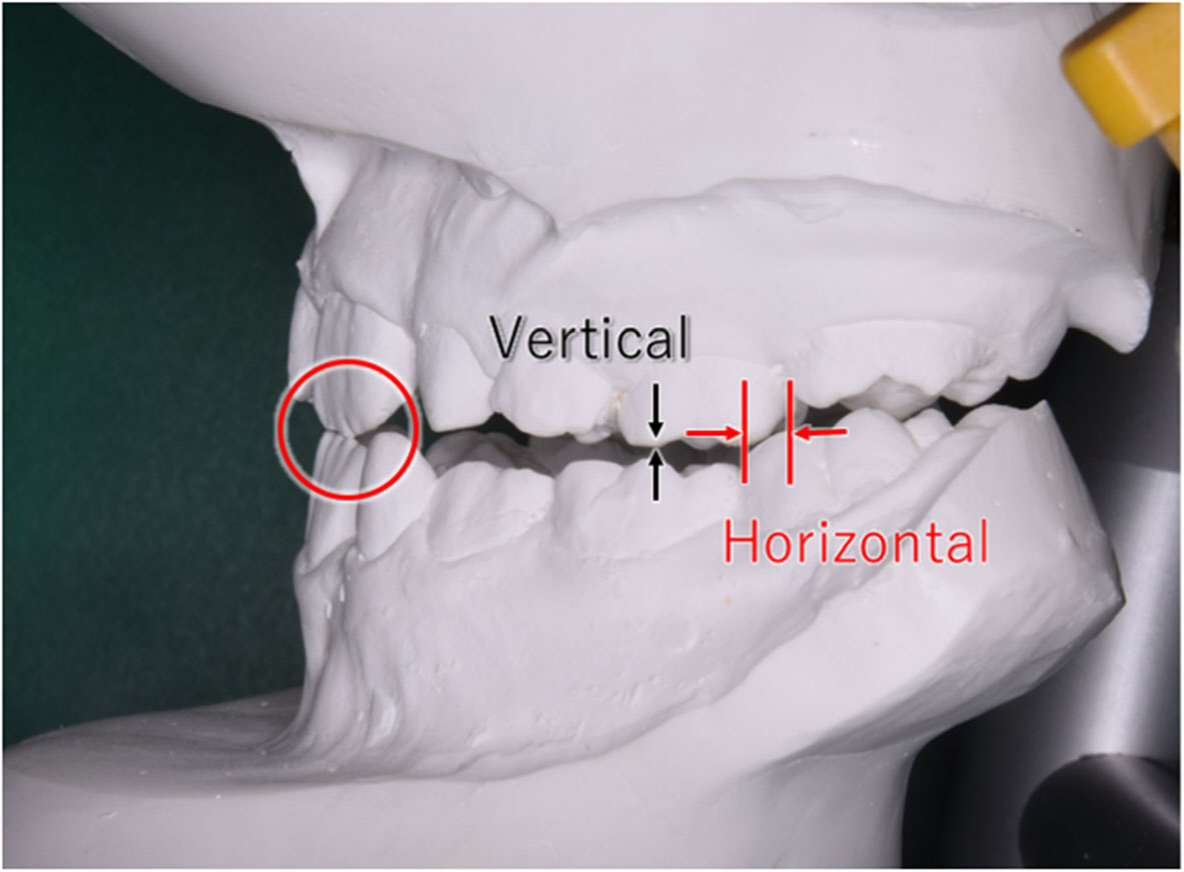
Horizontal and vertical distances on the mounted dental casts. The horizontal distance between the mesial contact point of the maxillary first molar and the mesial contact point of the mandibular first molar was measured bilaterally. The vertical distance between the mesiobuccal cusp of the maxillary second primary molar and the mesiobuccal cusp of the mandibular second primary molar was measured bilaterally

At the beginning of treatment, dental data were obtained from the models mounted in centric relation (CR) using Dowson’s technique (Fig. 1) [19]. The presence or absence of a functional shift was checked, and horizontal and vertical distances were measured with a digital ruler by YI as shown in Fig. 1. The distance between the mesial contact point of the maxillary first molar and the mesial contact point of the mandibular first molar was measured bilaterally for the horizontal distance. Three cases with mesial movement of the first molars due to early loss of the second primary molars were excluded from this analysis (resulting in 72 remaining subjects). The distance between the mesiobuccal cusps of the maxillary and mandibular second primary molars was measured for the vertical distance bilaterally. Vertical distances were measured for cases with a premature contact (*n* = 50).

**Fig. 2:**
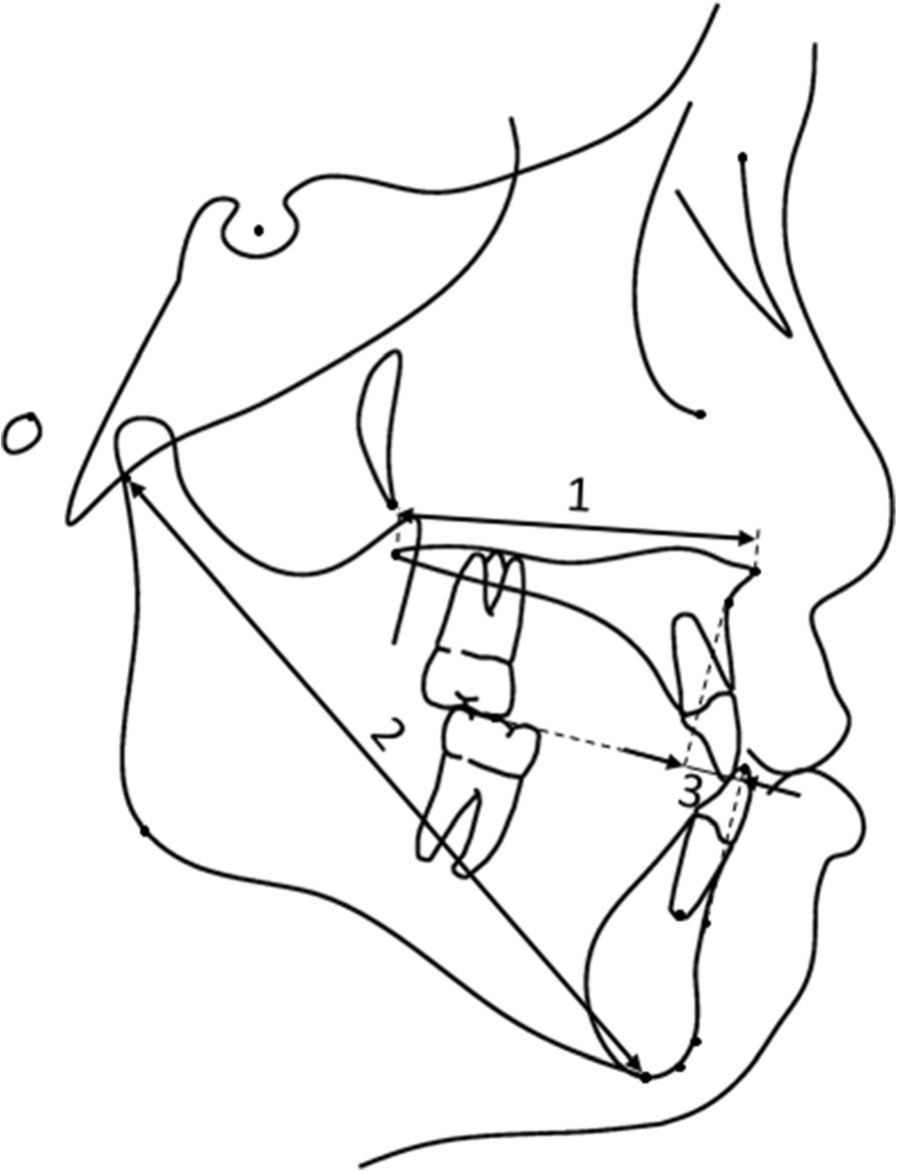
Linear measurements. (1) Maxillary length (ANS-PNS), (2) mandibular length (Ar-Me), and (3) Wits appraisal

**Fig. 3:**
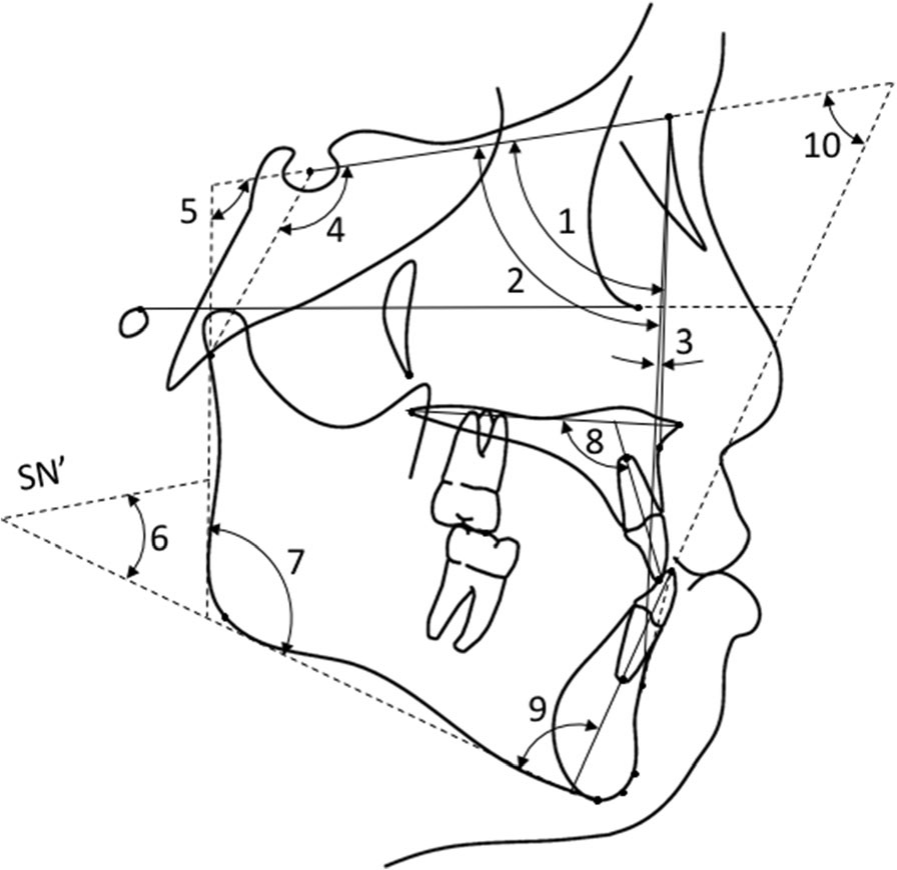
Angular measurements. (1) SNA angle, (2) SNB angle, (3) ANB angle, (4) SN to SN-Ar (SN-Ar), (5) SN to ramus plane angle (SN-Rm), (6) mandibular plane angle (SN-Md), (7) gonial angle, (8) upper incisor to palatal plane angle (U1-PP), (9) incisor mandibular plane angle (IMPA), and (10) Frankfort mandibular incisor angle (FMIA)

The initial lateral cephalograms were traced by YI who was blinded to the subjects’ stability grouping (Figs. 2 and 3). Intra-examiner reliability for the cast models and cephalometric films was assessed on 30 randomly selected sets that were measured and traced twice with a 1-month interval by the same examiner using the intraclass correlation coefficient.

### Statistical analysis

Statistical analysis was performed with SPSS Statistics ver. 24.0 (IBM Corp., Armonk, NY, USA).

Pearson’s chi-square test was used to compare the nominal qualitative variables (sex, age, presence of functional shift) of the stable and unstable groups. Mann-Whitney U tests were used to assess differences in the continuous variables (horizontal and vertical distances, cephalometric variables) between the stable and unstable groups because sample sizes were unbalanced and inadequate for the t-test. The significance level was set at 5%. For effect size (ES), w = 0.3 was used for the qualitative variables, and Cohen’s d = 0.8 was used for the continuous variables. Logistic regression analysis was used to determine which factors were most strongly associated with the results of early class III treatment. In analysis 1, the model included all factors that were evaluated as influential factors in the Mann-Whitney U test, such as the horizontal distance, mandibular length (Ar-Me), ANB, Wits, SN to ramus plane angle (SN-Rm), gonial angle, incisor mandibular plane angle (IMPA), and Frankfort plane to mandibular incisor angle (FMIA). In analysis 2, the horizontal distance was omitted from the factors of analysis 1.

## Results

Intraclass correlation estimates and their 95% confidence intervals were at acceptable levels, all above 0.90 for cephalometric measurements.

There was no significant difference in sex between the stable and unstable groups (*p* = .394) (Table 1).

**Table 1:** Cross tabulations of difference in sex and chi-square test

Sex	Stable	Unstable	*p*^a^
**Male**	30	8	.394
**Female**	25	12	

^a^ Chi-square test with yates correction (Statistical power was .738; Effect size w =0.3)

Although the mean age of the unstable group was higher than that of the stable group, there was no significant difference in the pretreatment age (*p* = .465) (Table 2).

**Table 2:** Descriptive statistics for age at the beginning of treatment

	Total			Stable			Unstable			U test
**Age**	n	X	IQR	n	X	IQR	n	X	IQR	*p*
	75	8.50	2.08	55	8.42	1.92	20	9.54	2.96	.465

Mann-Whitney’s U test between the two groups (Statistical power was .840; Effect size d =0.8) (*x* median, *IQR* interquartile range)

The numbers in the stable group with functional shift were much greater than the numbers in the unstable group without a functional shift. However, there was no significant difference in the anteroposterior functional shift between the two groups (*p* = .117) (Table 3).

**Table 3:** Cross tabulation of functional shift and chi-square test

Functional shift	Stable	Unstable	*p* ^a^
**Yes**	40	10	.117
**No**	15	10	

^a^ Chi-square test with Yates correction (Statistical power was .738; Effect size w = 0.3)

The findings for the horizontal and vertical distances are shown in Table 4. Significant differences were detected between the two groups for horizontal distance (*p* < .001). Although the mean value for vertical distance of the unstable group was greater than that of the stable group, no significant difference was observed (*p* = .326).

**Table 4:** Descriptive statistics and significance probabilities of horizontal and vertical variables

	Total			Stable			Unstable			U test
	*n*	X	IQR	*n*	X	IQR	*n*	X	IQR	*p*
**Horizontal**	72	1.43	1.73	53	1.00	1.35	19	2.50	2.10	.000*
**Vertical**	50	1.75	1.98	40	1.63	1.65	10	2.88	4.49	.326

Mann-Whitney’s U test between the stable group and the unstable group (Statistical powers were .840, and .581, respectively; Effect size d =0.8) (*x* median, *IQR* interquartile range, *: *p* < .05)

The cephalometric data revealed significant differences between the two groups in Ar-Me, Wits, SN-Rm, gonial angle, IMPA, and FMIA (Table 5). No marked difference was noted in the mandibular plane angle (SN-Md).

**Table 5:** Destrictive statistics and significance probabilities of cephalometric variables

	Total (*n* = 75)		Stable (*n* = 55)		Unstable (*n* = 20)		U test
	x	IQR	x	IQR	x	IQR	*p*
**PNS-ANS**	0.96	0.08	0.95	0.08	0.97	0.09	.101
**Ar-Me**	1.04	0.06	1.03	0.07	1.05	0.07	.024*
**SNA**	79.70	4.90	79.50	4.90	80.45	3.63	.260
**SNB**	79.80	3.90	79.40	4.10	80.40	4.43	.065
**ANB**	0.00	2.90	0.20	2.90	-0.90	2.28	.081
**Wits**	-6.60	3.40	-6.20	3.30	-7.70	2.75	.035*
**SN-Ar**	124.60	5.60	124.80	5.50	124.20	7.38	.573
**SN-Rm**	90.20	7.30	90.70	6.50	87.50	6.40	.049*
**SN-Md**	36.90	6.70	36.90	6.70	36.85	6.23	.480
**Gonial angle**	126.00	9.00	125.10	8.90	129.50	6.83	.024*
**U1-PP**	102.90	10.60	102.70	10.80	105.25	12.10	.253
**IMPA**	89.10	10.50	89.80	8.00	83.05	7.93	.009*
**FMIA**	60.90	9.50	59.30	9.60	65.05	9.43	.025*

Statistical powers were from 0.822 to 0.840; Effect size d =0.8 (*x* median, *IQR* interquartile range, *: *p* < .05)

### Logistic regression analysis

**Fig. 4:**
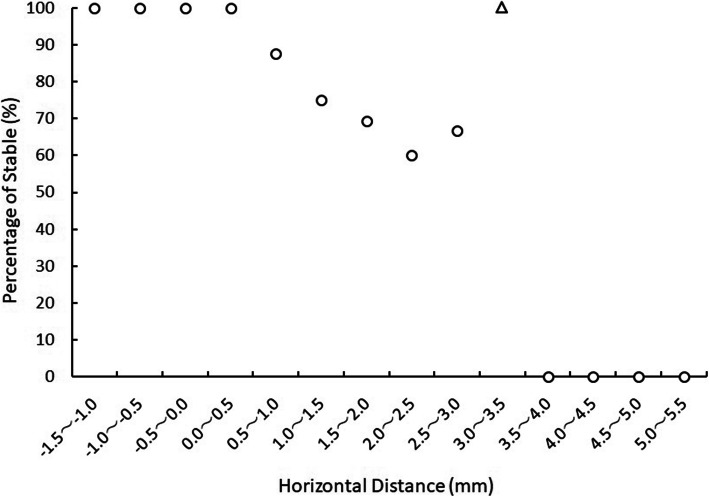
The percentage of stable cases relative to the pretreatment horizontal distance. The open triangle indicates an outlier. The occurrence of unstable cases started when the horizontal distance was more than 0.5 mm, and when this distance exceeded 3.5 mm, the percentage of stable cases was 0

Horizontal distance was the factor most strongly associated with the stability of early class III treatment in analysis 1. The odds ratio showed that when the horizontal distance increased by 1 mm, the number of unstable cases increased by 2.70 (95% confidence interval [CI] 1.45–5.01). Horizontal distance correctly predicted a stable versus unstable outcome in 79.2% of cases (57 of 72) (Table 6). The percentage of stable cases relative to the horizontal distance is shown in Fig. 4. The occurrence of unstable cases started when the horizontal distance was more than 0.5 mm. As the horizontal distance increased, the percentage of stable cases gradually decreased. When the horizontal distance was more than 3.5 mm, this percentage became 0.

**Table 6:** Logistic regression models using horizontal distance for the unstable group^a^

Independent variable	Logistic coefficient	Standard error	***p***	Odds ratio (95% confidence interval)	
**Horizontal (*n*** **= 72)**	0.992	0.317	.002	2.70	(1.45, 5.01)

^a^Dependent variable is Stable (=0) or Unstable (=1)

In analysis 2, SN-Rm, Gonial angle, and FMIA were detected as independent variables (Table 7). When SN-Rm increased by 1°, the odds ratio increased by 1.46; when the gonial angle increased by 1°, the odds ratio increased by 1.53; and when the FMIA increased by 1°, the odds ratio increased by 1.35. This model correctly predicted a stable versus unstable outcome in 78.7% of cases (59 of 75).

**Table 7:** Logistic regression models using cephalometric variables for the unstable group^a^

Independent variable	Logistic coefficient	Standard error	***p***	Odds ratio (95% Confidence interval)	
**SN-Rm**	-0.375	0.186	.044	1.46	(1.01, 2.10)
**Gonial angle**	-0.425	0.178	.017	1.53	(1.08, 2.17)
**FMIA**	-0.302	0.152	.048	1.35	(1.00, 1.82)

^a^ Dependent variable is Stable (=0) or Unstable (=1)

## Discussion

The most important finding of this study is that a significant difference in the stable and unstable groups was noted in the horizontal distance between the maxillary and mandibular molars from mounted casts. The unstable cases occurred when the horizontal distance was more than 0.5 mm, and the percentage of stable cases when this distance exceeded 3.5 mm was 0. This result means that the occasional case that present with a class III molar relation due to hypo-growth of the maxilla must become stable. Measuring the horizontal distance between the maxillary and mandibular molars in CR may be helpful for predicting the results of class III early treatment in daily clinical practice, even at the chairside.

Although the number of cases in the stable group with anteroposterior functional shift was greater than the number of cases in the unstable group without functional shift, there was no significant difference. Anteroposterior functional shift has been thought to be a positive factor in early class III treatment [16]. Most orthodontists believe that pseudo-class III cases are easier to treat than class III cases without anteroposterior functional shift; however, our findings call this belief into question.

In most previous studies [3–8], cephalometric factors alone were evaluated to predict the stability of early class III treatment. Possible reasons for this may be that most orthodontists assume that skeletal factors are associated with the outcome of class III cases. Additionally, the distance between the maxillary and mandibular molars of class III cases might be difficult to evaluate in the presence of a premature contact. Using mounted casts of class III cases enables the horizontal distance between the maxillary and mandibular molars to be measured, regardless of premature contacts. Hägg et al. [17] used cephalometric analysis to measure changes in the molar position at three stages (start of treatment, end of treatment, 8-year follow-up) and found significant differences between them; however, they were not evaluated as an associated pretreatment factor.

Regarding general factors, most orthodontists hypothesize that girls are more likely to achieve stability than boys. This may be due in part that Wolfe et al. [12] found that the anteroposterior discrepancies between the upper and lower jaws were larger in males than in females, and Alexander et al. [13] reported that the growth spurt in males was much greater than that in females. However, our findings indicated that there is no significant difference between the sexes. This result is consistent with those of a previous report that found no significant differences between the sexes for early class III treatment outcomes [6].

Most orthodontists also hypothesize that an early pretreatment age is more likely to have a favorable outcome than treatment at an older age. Indeed, maxillary protraction headgear has been reported to be more effective in younger patients [14, 15]. However, there were no significant differences in the pretreatment age between the two groups in the present study. This result is consistent with the findings of a meta-analysis showing no significant differences in the response to early treatment for class III malocclusion between starting at 7–10 years and 11–14 years of age [20].

Regarding cephalometric factors, mandibular length (Ar-Me) was associated with the stability outcome and was consistent with the results of previous studies [3–6]. The SN-Rm was also detected as a significant factor, a finding similar to those of previous studies concerning the association of the condylar axis inclination [2] and SN-Rm [3] with the outcome. Fudalej et al. [9] reported that the gonial angle was identified most frequently, and this factor was also indicated to be a significant predictor in the present study. Interestingly, significant differences were observed between the two groups in the ramus plane inclination and the gonial angle, but not in the mandibular plane angle. This may be because a small SN-Rm and an obtuse Gonial angle contribute to a normal mandibular plane angle. One of the most important points for clinicians to note is that it may be easy to overlook when the mandibular plane angle is normal, although cases with a small SN-Rm and large gonial angle may be unstable.

Mandibular incisors have been reported to be significantly retroclined in class III cases, except in the youngest patients [21]; therefore, the inclination of the mandibular incisors might be an important factor in predicting the outcome after the early mixed dentition stage. Our results indicated that there was a significant difference between the two groups in the IMPA and FMIA.

To summarize our findings relating to cephalometric factors, most of the data associated with stability were not related to maxillary measurements, contrary to what was reported by Ghiz et al. [6]. Because the mandible and maxilla have different types of ossification, cartilage, and sutural components, we suggest that the maxilla is more likely to respond to orthopedic force than the mandible. A previous study indicated that 25.0% of class III cases were related to retrusion of the maxilla, and 22.2% of the cases were related to a combination of maxillary retrusion and mandibular protrusion [21]. These findings therefore suggest that the remaining 52.8% of cases with mandibular protrusion might result in an unstable outcome.

### Strength and limitations

Although the long-term outcome of class III treatment was evaluated after comprehensive orthodontic treatment in most studies [4, 5, 7, 8], we evaluated stability before the comprehensive treatment. Therefore, our findings were able to show the exclusive effect of early class III treatment.

However, given this feature of our study, the mean age of 15 years for evaluation might be criticized as being too young for male patients. Because the flexion point of growth in Japanese boys is 13 years [22], which is younger than that in Caucasian boys (15 years) [23], the mean ages of the participants were thought to be acceptable for evaluating stability in Japanese boys. Growth can be expected to continue at a decreasing rate, and any remaining growth might influence the prediction of stability. Another interesting factor we would like to consider is information from patient’s family. We do often ask the patient and/or parents whether any family member had orthognathic surgery or protruded mandible, but this is just a questionnaire and not reliable quantitative information. The general public even describes it as protruding teeth when they see the compensated maxillary anterior teeth. Thus, if possible, if the patient’s parents permit us to take their cephalometric radiograph, it would be valuable information to predict the stability and possibility of the surgery. Another limitation of our study is that, even though our sample size was statistically sufficient, adding additional subjects with a wider range of severity may provide further information. Thus, future studies should observe the participants until growth is completed, after the completion of phase 2 therapy, and/or during retention with increased sample size to show the longitudinal results.

## Conclusions

1. Sex, pretreatment age, and anteroposterior functional shift were not related to early class III treatment stability.
2. The most influential factor in stability was the horizontal distance between the maxillary and mandibular molars in CR. If this distance exceeds 3.5 mm, the percentage of stable cases is 0.
3. Other suggested factors that might influence stability were the mandibular length (Ar-Me), Wits, SN-Rm, gonial angle, IMPA, and FMIA. Even if the mandibular plane is normal, it may be unstable in cases where the SN-Rm is small and the Gonial angle is large. Factors from the maxilla only were not related to stability.

## Data Availability

The datasets used and/or analyzed during the current study are available from the corresponding author on reasonable request.

## Abbreviations

CR: Centric relation
SN-Rm: SN to ramus plane angle
SN-Ar: SN to SN-Ar angle
U1-PP: Upper incisor to palatal plane angle
IMPA: Incisor mandibular plane angle
FMIA: Frankfort mandibular incisor angle
